# Lack of involvement of nucleotide excision repair gene polymorphisms in colorectal cancer

**DOI:** 10.1038/sj.bjc.6601061

**Published:** 2003-07-15

**Authors:** R Mort, L Mo, C McEwan, D W Melton

**Affiliations:** 1 Sir Alastair Currie Cancer Research UK Laboratories, Molecular Medicine Centre, University of Edinburgh, Western General Hospital, Crewe Road, Edinburgh EH4 2XU, UK

**Keywords:** DNA damage, DNA repair, loss of heterozygosity, xeroderma pigmentosum

## Abstract

DNA repair has an essential role in protecting the genome from damage by endogenous and environmental agents. Polymorphisms in DNA repair genes and differences in repair capacity between individuals have been widely documented. For colorectal cancer, the loss of mismatch repair gene activity is a key genetic determinant. Nucleotide excision repair (NER), recombination repair (RR) and base excision repair (BER) pathways have critical roles in protection against other cancers, and we wished to investigate their role in colorectal cancer. We have compared the frequency of polymorphisms in the NER genes, *XPD*, *XPF*, *XPG*, *ERCC1*; in the BER gene, *XRCC1*; and in the RR gene, *XRCC3*; in colorectal cancer patients and in a control group. No significant associations were found for any of the NER gene polymorphisms or for the *XRCC1* polymorphism. The C allele (position 18067) of the *XRCC3* gene was weakly but significantly associated with colorectal cancer (odds ratio 1.52, 95% confidence interval 1.04–2.22, *P*=0.03). For all patients who were heterozygous for any of the repair genes studied, tumour tissue was investigated for loss of heterozygosity (LOH). Only one example of LOH was found for all the genes examined. From the association and LOH data, we conclude that these genes do not have an important role in protection against colorectal carcinogenesis.

Several complementary DNA repair mechanisms have evolved to protect the genome from DNA damage caused by endogenous or environmental agents, which could lead to mutations and carcinogenesis (Friedberg, 2001). DNA repair capacity varies between individuals in the general population (for a review, see Berwick and Vineis, 2000). An increasing number of DNA repair gene polymorphisms are being described and their involvement in carcinogenesis is being investigated. For colorectal cancer, the importance of mutations in mismatch repair (MMR) genes has been extensively documented. MMR gene defects account for 15% of sporadic colorectal cancer, and germline mutations in MMR genes are the cause of hereditary nonpolyposis colon cancer (for a review, see Jiricny and Nystrom-Lahti, 2000). The role of additional low-penetrance genes in colorectal cancer susceptibility has been recently reviewed (de Jong *et al*, 2002). We wished to investigate the hypothesis that alterations in other DNA repair pathways were also important genetic determinants of colorectal carcinogenesis.

The nucleotide excision repair (NER) pathway deals with UV light-induced DNA damage (for a review, see Wood, 1997). In the inherited disorder, xeroderma pigmentosum, NER deficiency results in a 1000-fold increased incidence of skin cancer, but also a 20-fold increase in internal tumours (Friedberg *et al*, 1995), indicating that NER is also important in the repair of endogenous DNA damage. Indeed, the digestive tract contains materials such as lipid peroxidation by-products that can react with DNA to generate bulky adducts that are recognised by NER (Friedberg *et al*, 1995). Amino-acid variants in NER genes are common in the general population (Shen *et al*, 1998) and some, such as the *XPD* exon 23 polymorphism, have been associated with reduced DNA repair capacity (Lunn *et al*, 2000; Qiao *et al*, 2002). We have reported a significant association between polymorphisms in exons 6, 22 and 23 of the *XPD* gene and melanoma in patients under 50 (Tomescu *et al*, 2001). Significant association between NER gene polymorphisms and other cancers, but not colorectal, have also been reported: glioma (Chen *et al*, 2000); lung (Chen *et al*, 2002; Park *et al*, 2002); squamous cell carcinoma of the head and neck (SCCHN; Sturgis *et al*, 2002).

The *XRCC1* gene is involved in the repair of single-strand DNA breaks and in base excision repair (BER) of damaged bases caused by endogenous and exogenous oxidants, including tobacco smoke. *XRCC1* polymorphisms have been associated with SCCHN (Sturgis *et al*, 1999), pancreatic adenocarcinoma (Duell *et al*, 2002), lung cancer (Chen *et al*, 2002) and bladder cancer (Stern *et al*, 2002). There is also a single report of a significant association with colorectal cancer (reviewed by de Jong *et al*, 2002). The *XRCC3* gene, a paralogue of RAD51, is involved in recombination repair (RR) and is required for genome stability (Griffin *et al*, 2000). *XRCC3* polymorphisms have been associated with melanoma (Winsey *et al*, 2000), bladder cancer (Matullo *et al*, 2001a; Stern *et al*, 2002) and SCCHN (Shen *et al*, 2002).

Loss of heterozygosity (LOH) of tumour suppressor genes, such as p53, *APC* and *BRCA1*, is an important step in carcinogenesis. LOH for NER genes has been reported as a common occurrence in a range of carcinomas (Takebayashi *et al*, 2001), and we also wished to study LOH of NER genes in our colorectal cancer samples.

In this study, we have compared the frequency of polymorphisms in the NER genes (*XPD*, *XPF*, *XPG*, *ERCC1*), and in *XRCC1* and *XRCC3* in colorectal cancer patients and a control group. Furthermore, for patients heterozygous at any of these loci, we have looked for LOH in a biopsy of tumour tissue.

## MATERIALS AND METHODS

### Subjects

Subjects were colorectal cancer patients (mean age 69 years) attending the Western General Hospital, Edinburgh, UK. Biopsy material, from cancerous and adjacent noncancerous tissue, was collected between 1994 and 1997 by Professor Andrew Wyllie for histology and DNA extraction. At the same time, control blood samples were selected entirely at random from donors to the Scottish National Blood Transfusion Service (mean age of donors is 42 years), and DNA was extracted as described (Tomescu *et al*, 2001).

### PCR and RFLP assays

The polymorphisms studied are shown in Table 1

**Table 1:** Details of RFLPs studied and fragment sizes

**Gene/exon**	**Enzyme**	**Polymorphism**	**Position^a^**	**Genotype**	**Fragment sizes (bp)**
*XPD* exon 6	*Hinf*I	C to A	22541	CC	288 (no cut)
				CA	288+206+82
				AA	206+82
*XPD* exon 22	*Fok*I	C to T	35326	CC	229 (no cut)
				CT	229+135+94
				TT	135+94
*XPD* exon 23	*Pst*I	A to C	35931	AA	234+110
				AC	234+172+110+62
				CC	172+110+62
*ERCC1* exon 4	*Bsr*DI	G to A	19007	GG	252 (no cut)
				GA	252+179+73
				AA	179+73
*XPG* exon 15	*Nla*III	G to C	3507	GG	271 (no cut)
			(cDNA)	GC	271+227+44
				CC	227+44
*XPF* exon 11	*Alw*NI	T to C	30028	TT	442+267
				TC	709+442+267
				CC	709 (no cut)
*XRCC1* exon 17	*Stu*I	G to A	36189	GG	343+158
				GA	501+343+158
				AA	501 (no cut)
*XRCC3* exon 7	*Nla*III	C to T	18067	CC	346 (no cut)
				CT	346+242+104
				TT	242+104

a Nucleotide positions are from the GenBank entries: *XPD*, L47234; *ERCC1*, M63796; *XPG*, NM_000123; *XPF*, L76568; *XRCC1*, L34079; *XRCC3*, AF037222.

. All, apart from the *XPG* exon 15 polymorphism (Emmert *et al*, 2001), were originally described by Shen *et al* (1998). Polymorphisms were chosen for study because, in each case, the variant allele was common and the single-nucleotide change resulted in the gain, or loss, of a restriction site so that the polymorphism could be easily typed by PCR and RFLP analysis. In addition, significant associations between some of these polymorphisms and cancer have previously been reported. Details of the RFLPs are shown in Table 1. Details of the PCR products, primers and cycle conditions used are shown in Table 2

**Table 2:** Details of PCR products for polymorphism analysis

**Product**	**Primer sequence**^a^	**Size (bp)**	**Conditions**
*XPD* exon 6	(F)TGTCCAAAACCCCAGCCAGCTG	288	30 cycles: 94°C 1 min, 69°C 1 min, 72°C 30 s
	(R)CAGGGGTCAGGGAGGCTGCCTG		
*XPD* exon 22	(F)AATGACCTTCTGTCCCTGGCCTGCG	229	35 cycles: 94°C 1 min, 72°C 30 s
	(R)AGAAGCTCAGCCTGGGAGGGTGCCG		
*XPD* exon 23	(F)TCAAACATCCTGTCCCTACTGGCCAT	344	35 cycles: 94°C 1 min, 67°C 1 min, 72°C 30 s
	(R)CTGCGATTAAAGGCTGTGGACGTGAC		
*ERCC1* exon 4	(F)TCATCCCTATTGATGGCTTCTGCCC	252	35 cycles: 94°C 1 min, 69°C 1 min, 72°C 30 s
	(R)GACCATGCCCAGAGGCTTCTCATAG		
*XPG* exon 15	(F)GACCTGCCTCTCAGAATCATC	271	35 cycles: 94°C 1 min, 62°C 1 min, 72°C 1 min
	(R)CCTCGCACGTCTTAGTTTCC		
*XPF* exon 11	(F)TCTCCATGTCCCGCTACTAC	709	35 cycles: 94°C 1 min, 67°C 1 min, 72°C 1 min
	(R)GCAGGCACAGGCAAGTTCAA		
*XRCC1* exon 17	(F)GTCAGTGCTGATTCCCTGATGTGCC	501	35 cycles: 94°C 1 min, 69°C 1 min, 72°C 1 min
	(R)CAGATCTCTGACGGAGGTGCCCAGC		
*XRCC3* exon 7	(F)GCTCGCCTGGTGGTCATCGACTC	346	35 cycles: 94°C 1 min, 69°C 1 min, 72°C 1 min
	(R)CTGTCACCTGGAAGAGCACAGTCC		

a Primer sequences were derived from the GenBank entries.

. Genomic DNA (∼100 ng) was amplified in a 50 *μ*l reaction volume containing 300 ng of each primer and 2.5 U *Taq* DNA polymerase (Promega, UK) in 50 mM KCl, 1.5 mM MgCl_2_, 10 mM Tris-HCl pH 8.3, 0.45% Triton X-100, 0.45% Tween 20, 0.4 mM Na_2_EDTA, 0.1 mM dNTPs.

### Statistical analysis

We first investigated whether the observed genotype distributions at each locus were consistent with a Hardy–Weinberg equilibrium. Having confirmed that this was the case, we assumed that alleles were independent at each locus, and compared allele counts among cases and controls. A *χ*^2^-test with Yates' correction was used to give a conservative test for the significance of any association between a polymorphism and colorectal cancer.

## RESULTS

**Figure 1:**
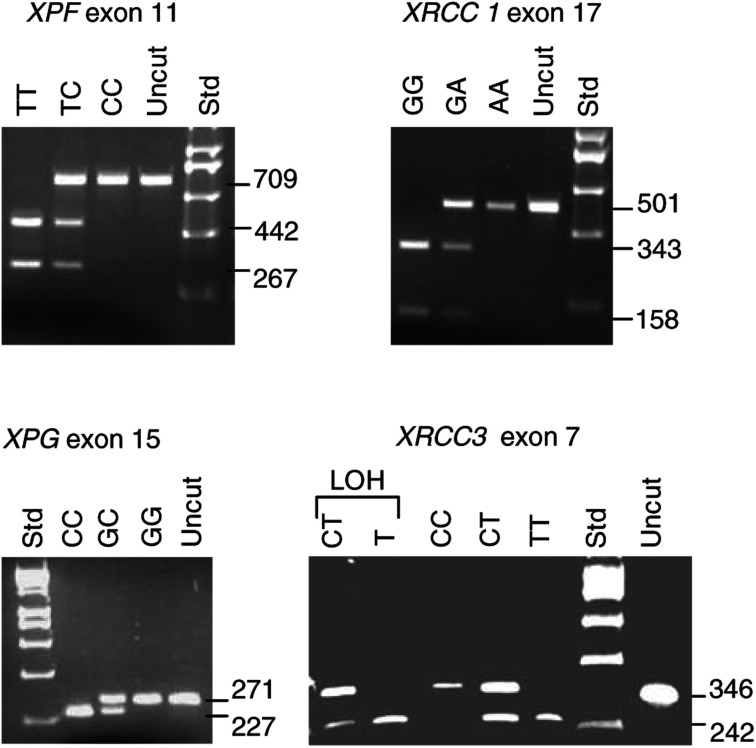
RFLP analysis of DNA repair gene polymorphisms. The patterns obtained for the polymorphisms in *XPF* exon 11, *XRCC1* exon 17, *XPG* exon 15 and *XRCC3* exon 7 are indicated. In each case, the sizes (bp) of the fragments generated for each genotype by restriction of the PCR products are shown, along with the uncut PCR product and a molecular size standard (Std). Details of the digests used and fragment sizes are given in Table 1. Note that the 44 bp fragment for the *XPG* assay and the 104 bp fragment for the *XRCC3* assay are not resolved on the gels used. For the *XRCC3* assay, LOH in a colorectal tumour sample is also shown. Normal tissue from the patient is CT, but the tumour is T.

Initially, we set out to genotype a minimum of 40 patient and control samples for the eight DNA repair gene RFLPs shown in Table 1. Where less genotypes than this are reported for each sample group, it is because some genotyping reactions failed. In the one instance (*XRCC3* exon 7), where a significant association was found during this first round of genotyping, additional patient and control samples were then genotyped to increase the power of the statistical analysis. All the genotyping data obtained from the patient and control samples analysed are presented below. The patterns obtained for *XPF* exon 11, *XPG* exon 15, *XRCC1* exon 17 and *XRCC3* exon 7 are illustrated in Figure 1

. The patterns for the *XPD* (exons 6, 22 and 23) and *ERCC1* exon 4 polymorphisms have been described previously (Tomescu *et al*, 2001). For each polymorphism investigated in the control and patient groups, the observed genotype distributions were compared with the expected frequencies under the Hardy–Weinberg equilibrium. In each case, there were no significant deviations from the expected values. We therefore assumed independence of alleles at these loci and compared allele counts, rather than genotypes, in patient and control groups because of the resulting increased power of the statistical analysis.

### Lack of association between NER gene polymorphisms and colorectal cancer

No significant associations were found between the *XPD* polymorphisms (exons 6, 22 and 23) and colorectal cancer (Table 3

**Table 3:** Lack of association of *XPD* polymorphisms and colorectal cancer

	***XPD*** exon 6		***XPD*** exon 22		***XPD*** exon 23	
	**A**	**C**	**C**	**T**	**A**	**C**
Patients^a^	41	49	56	34	50	40
Controls^b^	56	86	56	32	60	34
	OR 1.28, *P*^c^=0.43		OR 0.94, *P*^c^=0.97		OR 0.71, *P*^c^=0.32	
	(95% CI 0.73–2.27)		(95% CI 0.49–1.81)		(95% CI 0.38–1.34)	

a Patients: *n*=45.

b Controls: exon 6, *n*=71; exon 22, *n*=44; exon 23, *n*=47.

c *χ*^2^ analysis with Yates', correction.

). Similarly, no significant associations were found for *XPF* exon 11, *XPG* exon 15 and *ERCC1* exon 4 (Table 4

**Table 4:** Lack of association of NER polymorphisms and colorectal cancer

	***XPF*** exon 11		***XPG*** exon 15		***ERCC1*** exon 4	
	T	**C**	**C**	**G**	**G**	**A**
Patients^a^	61	19	13	67	38	52
Controls^b^	57	9	22	58	58	86
	OR 0.51, *P*=0.18		OR 0.51, *P*=0.13		OR 1.08, *P*=0.87	
	(95% CI 0.19–1.30)		(95% CI 0.22–1.18)		(95% CI 0.61–1.92)	

a Patients: *XPF* and *XPG, n*=40; *ERCC1*, *n*=45.

b Controls: *XPF*, *n*=33; *XPG*, *n*=40; *ERCC1*, *n*=72.

).

### Association between *XRCC3* exon 7 polymorphism and colorectal cancer

Although no significant association with colorectal cancer was found for the *XRCC1* exon 17 polymorphism (Table 5

**Table 5:** Association of *XRCC3* exon 7 C allele and colorectal cancer

	***XRCC1*** exon 17		***XRCC3*** exon 7	
	**G**	**A**	**C**	**T**
Patients^a^	40	40	161	85
Controls^b^	38	28	142	114
	OR 0.74, *P*=0.46		OR 1.52, *P*=0.03	
	(95% CI 0.36–1.50)		(95% CI 1.04–2.22)	

a Patients: *XRCC1*, *n*=40; *XRCC3*, *n*=123.

b Controls: *XRCC1*, *n*=33; *XRCC3*, *n*=128.

), a significant association was found for the *XRCC3* polymorphism in the first batch of 40 samples genotyped. This association was maintained as the sample size of patient and control groups was increased to over 120, such that the *XRCC3* exon 7 C allele was significantly over-represented in the patient group (odds ratio (OR) 1.52, 95% confidence interval (CI) 1.04–2.22, *P*=0.03).

### No LOH at NER gene loci in colorectal cancer

Where normal tissue biopsied from colorectal cancer patients showed heterozygosity at any of the DNA repair gene loci genotyped, the tumour tissue from the same patients was analysed for LOH. The numbers of heterozygous loci examined in the patients were: 16 for *XPD* exon 6; 21 for *XPD* exon 22; 17 for *XPD* exon 23; 16 for *ERCC1* exon 4; 16 for *XPF* exon 11; 13 for *XPG* exon 15; 22 for *XRCC1* exon 17; 28 for *XRCC3* exon 7. From these 149 heterozygous loci examined, only a single example of LOH was found. This occurred for an *XRCC3* exon 7 CT heterozygote where the C allele was absent in the tumour (Figure 1).

## DISCUSSION

We have investigated the hypothesis that NER gene polymorphisms might predispose to colorectal cancer because some forms of the encoded proteins may be less efficient at repairing DNA damage arising from exposure of the gut epithelium to genotoxic compounds in the lumen. NER is known to be active against a range of bulky DNA lesions in addition to its main role in the repair of UV-induced DNA damage. In a small study, we have previously shown a significant association between melanoma and three polymorphisms in the *XPD* gene (exon 6 (position 22541) A allele; exon 22 (position 35326) C allele; exon 23 (position 35931) A allele) that did not extend to markers flanking the *XPD* gene (Tomescu *et al*, 2001). A similar association for the *XPD* exons 6 and 23 polymorphisms has been found in some other, but not all, skin cancer studies (for a review, see Benhamou and Sarasin, 2002). In some studies, the protein encoded by the *XPD* exon 23A allele (Lys → Gln change) has been associated with reduced DNA repair activity (for a review, see Benhamou and Sarasin, 2002). The *XPD* exon 23 polymorphism has also been associated with lung cancer (Chen *et al*, 2002) and an exon 10 polymorphism with SCCHN (Sturgis *et al*, 2002). A polymorphism in the 3′ UTR of *ERCC1* (not the exon 4 RFLP studied here) has been associated with glioma (Chen *et al*, 2000) and SCCHN (Sturgis *et al*, 2002) and two XPA polymorphisms are associated with lung cancer (Park *et al*, 2002). Some of the polymorphisms we have studied lead to a change in the encoded protein (*XPD* exon 23 Lys-Gln; XPG exon 15 Asp-His; XRCC3 exon 7 Thr-Met), others are silent (*XPD* exons 6 and 22; XPF exon 11; ERCC1 exon 4; XRCC1 exon 17). A significant association between a protein variant and cancer only indicates that the change might be causative. An association with a silent change indicates that the causative change is closely linked, but remains to be identified. No significant association was found between any of the NER gene polymorphisms studied and colorectal cancer. While this does not exclude the possibility that NER gene polymorphisms might make a minor contribution to genetic predisposition to colorectal cancer, a much larger study than the present one would be required to identify such a minor risk factor.

Similarly, although polymorphisms in exons 6 and 10 of the *XRCC1* gene, that is involved in BER and the repair of single-strand DNA breaks, have been associated with colorectal cancer (reviewed in de Jong *et al*, 2002) and a range of other internal cancers (Sturgis *et al*, 1999; Chen *et al*, 2002; Duell *et al*, 2002; Stern *et al*, 2002), no association was seen in our study between the *XRCC1* exon 17 polymorphism and colorectal cancer.

A significant association was found for the recombinational repair gene, *XRCC3*, where the exon 7 C (position 18067, Thr) allele showed a modest (OR 1.52), but significant (*P*=0.03) over-representation in 123 colorectal cancer patients compared to 128 controls. This *XRCC3* exon 7 polymorphism has previously shown a significant association with melanoma (Winsey *et al*, 2000), bladder cancer (Matullo *et al*, 2001a) and SCCHN (Shen *et al*, 2002). However, in these studies, it was the variant T (Met) allele that was over-represented in the cancer patients and this allele has also been associated with a reduced DNA repair phenotype (Matullo *et al*, 2001b).

Conflicting data from association studies between DNA repair gene polymorphisms and cancer susceptibility, or DNA repair capacity, are not unusual (e.g. see Benhamou and Sarasin, 2002, for a review of the *XPD* data) and many probably result from insufficient sample sizes or inappropriate choice of controls in some studies. However, Nelson *et al* (2002) have considered how studies on DNA repair gene polymorphisms can produce reliable, but opposite, associations in different types of cancer. The example they considered was the *XRCC1* Arg399Gln polymorphism. They suggest that the outcome of a repair variant in a cell may depend on the selection pressures exerted on that cell. If the polymorphism results in reduced repair efficiency, the resultant increased levels of DNA damage may lead to an increased rate of apoptosis. Such a polymorphism would be associated with increased cancer risk in nondividing, or ‘apoptosis-abrogated’ cells and reduced cancer risk in cells with the capacity for apoptosis. Nelson *et al* (2002) also highlight that high UV exposure to the skin results in p53 mutant cells which do not apoptose. The variant *XRCC3* exon 7 T allele with reduced repair capacity could result in an increased cancer risk in the skin (by the fixation of mutations in p53 mutant cells) and a protective effect in the gut (by more efficient apoptosis).

The mechanism for the reduced repair capacity associated with the variant *XRCC3* exon 7 T allele is unknown. It may alter the protein's interaction with RAD51, but could also affect other aspects of the role of XRCC3. This highlights the main difficulty with studies designed to look at single polymorphisms without knowledge of their functional significance. Without detailed knowledge of XRCC3's roles in DNA repair, it is hard to evaluate the significance of such studies, or exclude a role for confounding factors. In the same way as we have done for *XPD* and melanoma (Tomescu *et al*, 2001), study of *XRCC3* flanking markers will be required to rule out roles for linkage disequilibrium and population stratification in the association found.

Our study suggested that LOH of NER genes and *XRCC1* and *XRCC3* does not play an important role in colorectal carcinogenesis. Indeed, only a single example of LOH was found (for *XRCC3*) in 149 heterozygous loci examined. In addition to reporting a high rate of LOH for NER genes in ovarian and lung cancer, Takebayashi *et al* (2001) reported LOH for NER genes in 16.7% of colon carcinomas. Closer examination of their data reveals that this equates to LOH for *XPE* in a single patient and LOH for *XPB* and *XPE* in a second patient, from a total of 12 patients examined and eight NER genes typed (44 out of 96 assays were noninformative). Thus, the two studies are in agreement that the frequency of LOH for NER genes is low in colorectal cancer.

In conclusion, association and LOH studies suggest that NER genes are not important genetic determinants for colorectal carcinogenesis. A modest association between colorectal cancer and a polymorphism in the recombination repair gene *XRCC3* was found, but additional studies will be required to evaluate its importance.
